# Influence of the extracellular matrix on endogenous and transplanted stem cells after brain damage

**DOI:** 10.3389/fncel.2014.00219

**Published:** 2014-08-19

**Authors:** Lars Roll, Andreas Faissner

**Affiliations:** ^1^Department of Cell Morphology and Molecular Neurobiology, Ruhr-University BochumBochum, Germany; ^2^International Graduate School of Neuroscience, Ruhr-University BochumBochum, Germany

**Keywords:** brain damage, stem cells, stem cell niche, reactive gliosis, extracellular matrix

## Abstract

The limited regeneration capacity of the adult central nervous system (CNS) requires strategies to improve recovery of patients. In this context, the interaction of endogenous as well as transplanted stem cells with their environment is crucial. An understanding of the molecular mechanisms could help to improve regeneration by targeted manipulation. In the course of reactive gliosis, astrocytes upregulate Glial fibrillary acidic protein (GFAP) and start, in many cases, to proliferate. Beside GFAP, subpopulations of these astroglial cells coexpress neural progenitor markers like Nestin. Although cells express these markers, the proportion of cells that eventually give rise to neurons is limited in many cases *in vivo* compared to the situation *in vitro*. In the first section, we present the characteristics of endogenous progenitor-like cells and discuss the differences in their neurogenic potential *in vitro* and *in vivo*. As the environment plays an important role for survival, proliferation, migration, and other processes, the second section of the review describes changes in the extracellular matrix (ECM), a complex network that contains numerous signaling molecules. It appears that signals in the damaged CNS lead to an activation and de-differentiation of astrocytes, but do not effectively promote neuronal differentiation of these cells. Factors that influence stem cells during development are upregulated in the damaged brain as part of an environment resembling a stem cell niche. We give a general description of the ECM composition, with focus on stem cell-associated factors like the glycoprotein Tenascin-C (TN-C). Stem cell transplantation is considered as potential treatment strategy. Interaction of transplanted stem cells with the host environment is critical for the outcome of stem cell-based therapies. Possible mechanisms involving the ECM by which transplanted stem cells might improve recovery are discussed in the last section.

## Introduction

Regeneration of the adult central nervous system (CNS) after damage is limited in mammals. This causes severe problems for patients who suffer from CNS lesions or stroke. Regeneration of the peripheral nervous system is more effective, differences in the cellular response are attributed to this discrepancy (Brosius Lutz and Barres, 2014). Several approaches have been tested in animal models to improve functional recovery of patients, for instance neutralization of inhibitory factors by injection of blocking antibodies or by enzymatic degradation (Zhao et al., 2013). Transplantation of stem cells are another promising strategy and effort is made to examine their effect on regeneration in clinical trials (Savitz et al., 2014). For an efficient treatment, an understanding of the cellular and molecular mechanisms underlying the observed limitations is of interest.

In the first section we describe the effect of CNS damage on astrocytes that become reactive and in many cases start to re-express neural progenitor markers. The second section is focused on the extracellular matrix (ECM) and factors in this matrix expressed by these astrocytes and other cell types. The third section is dedicated to the question how transplanted stem cells can interact with the host and by which mechanisms they might improve regeneration.

## Lesions of the central nervous system activate endogenous progenitor-like cells

**Figure 1:**
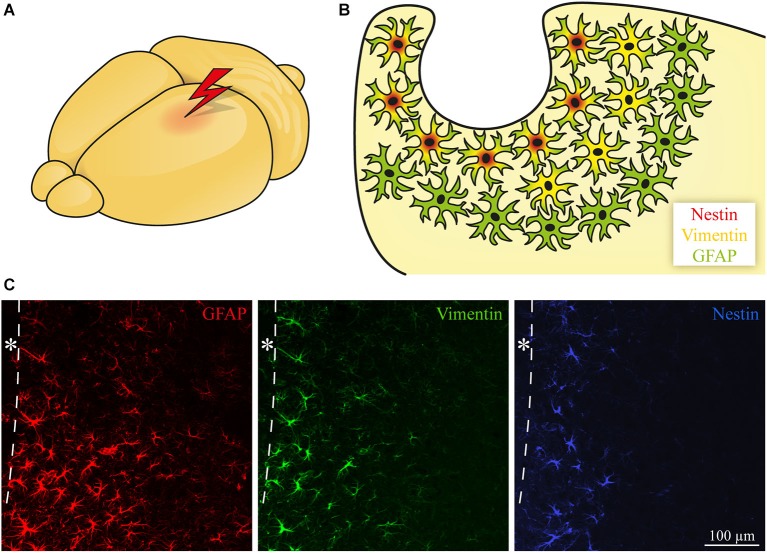
**Subtypes of reactive astroglia. (A, B)** Astrocytes become reactive following CNS damage. In addition to GFAP upregulation, some of the cells start to express progenitor markers like Vimentin and Nestin. The position of the cells in relation to the lesion determines the expression pattern with Nestin near the lesion, Vimentin in a broader area and GFAP with a widespread upregulation. **(C)** Immunohistochemical staining of GFAP (red), Vimentin (green), and Nestin (blue, in an adjacent slice) expression after focal laser lesion of the mouse visual cortex. The dashed line depicts the border to the lesion core; * lesion core.

After stroke or lesion, astrocytes, microglia, and other cell types of the immune system are activated in a process called reactive gliosis (reviewed by Donnelly and Popovich, 2008; Burda and Sofroniew, 2014). Astrocytes change their morphology and subpopulations of them upregulate markers typical of immature neural progenitors during development (Figure 1). In severe cases, a glial scar is formed by astrocytes that intermingle with fibroblasts before they segregate (Bundesen et al., 2003). In the following first section of the review, we want to give a short overview of the markers these astroglial subtypes express, of their spatial distribution and origin, and of the neurogenic potential these cells might have *in vivo*.

### Progenitor marker expression after CNS damage

Astrocytes become reactive after CNS damage and typically express the intermediate filament Glial fibrillary acidic protein (GFAP). In addition, other markers like the intermediate filaments Vimentin and Nestin are coexpressed (Pekny and Nilsson, 2005). Vimentin is expressed in radial glia during development (Bignami et al., 1982), whereas Nestin is the prototypical neural stem/progenitor cell marker (Lendahl et al., 1990). Depending on the time point after lesion, additional markers for immature glia like Brain lipid-binding protein (BLBP), the DSD-1 epitope on members of the receptor protein tyrosine phosphatase (RPTP) β family, and Tenascin-C (TN-C) are expressed (Robel et al., 2011).

The function of GFAP and Vimentin in regeneration is discussed (Brenner, 2014). For example, knockout mice deficient for GFAP and Vimentin showed reduced hypertrophy of astrocytes, improved synaptic restoration after entorhinal cortex lesion (Wilhelmsson et al., 2004), and supported integration of cells transplanted into the retina (Kinouchi et al., 2003) or into the hippocampus (Widestrand et al., 2007). In contrast, an increased lesion volume was found in GFAP/Vimentin double knockout mice after stroke, whereas single knockouts had no effect on the lesion volume (Li et al., 2008). Different GFAP isoforms are described for human and mice, but especially for mice there is no clear correlation of certain isoforms with stem/progenitor cells (Kamphuis et al., 2012). The role of TN-C and other ECM components in regeneration is discussed in the second section.

### Astroglial subtypes are arranged in distinct areas

The different markers mentioned above are not uniformly expressed by all astrocytes. Instead, subpopulations express different combinations. Here it was observed that the cells show a specific spatial distribution. For example, focal laser lesions in the adult mouse visual cortex (Roll et al., 2012) induce GFAP upregulation in a wide area, whereas the progenitor markers Vimentin and even more extremely Nestin are restricted to an area near the lesion core (Figure 1). Interestingly, astrocytes (type B cells) are the stem cells of the subventricular zone (SVZ) and are also positive for Nestin and Vimentin (Doetsch et al., 1997, 1999). Differences in marker expression may reflect the potential of the cells to form other neural cell types. It was described that multipotent neural stem/progenitor cells that give rise to neurons, astrocytes, and oligodendrocytes *in vitro* appear in the brain following stab wound (Buffo et al., 2008), laser lesion (Sirko et al., 2009), and in other lesion models (Sirko et al., 2013). As shown by differential marker expression, reactive astrocytes are a heterogeneous population with respect to the distance of a cell to the lesion. Additionally, astrocytes are also heterogeneous regarding morphology, function, CNS region, and severity of the lesion (reviewed by Anderson et al., 2014).

### Different origins of multipotent cells after CNS damage

An obvious question regarding multipotent stem/progenitor cells in the damaged adult brain is the origin of those cells. Are adult stem cells attracted from the stem cells niches like the SVZ and migrate to the lesion site, or are local astrocytes induced to de-differentiate on-site? An argument for activation of local cells in focal laser lesions of the visual mouse cortex is the distinct spatial distribution of markers like GFAP, Vimentin, and Nestin. A similar finding of Nestin-expressing cells in a distinct pattern was made in the spinal cord after hemitransection and was also interpreted as local activation (Lang et al., 2004). Re-expression of the ECM molecule TN-C, which is expressed during development and later downregulated in the adult cortex, is also restricted to astrocytes located near the lesion (McKeon et al., 1991; Roll et al., 2012). It can be assumed that gradients of signaling molecules with high concentrations near the lesion and decreasing levels in the periphery influence the cell fate and result in the observed regional differences. Indeed, fate mapping studies by Buffo et al. (2008) showed that stab wounds activate local astrocytes in the cortex that are multipotent *in vitro*. Multipotent cells that give rise to neurons, astrocytes, or oligodendrocytes can be found in the developing nervous system. Different tripotential, but also subtypes of bipotential glial precursors have been described there. For example, glial restricted precursors (GRPs) that produce oligodendrocytes and astrocytes, O2A cells that give rise to oligodendrocytes and type-2 astrocytes, and others are distinguished according to their potential *in vivo* and *in vitro* and to their marker expression (Liu and Rao, 2004). The proteoglycan Neuron-glial antigen 2 (NG2) is associated with glial precursors during development, therefore the contribution of NG2-positive cells present in the adult CNS after damage is discussed (Han et al., 2004; Komitova et al., 2011). In the spinal cord, it has been shown that ependymal cells contribute significantly to newly formed astrocytes and show multilineage potential (Barnabé-Heider et al., 2010). To what extent cells after damage only share similarities or if they acquire a cell fate that is indeed identical to those developmental populations is hard to determine. Depending on the severity, in addition to a local response cells from the adult stem cell niches are activated (Shimada et al., 2010). A stem cell response in terms of an increased SVZ size (Thored et al., 2006) and attraction of neuroblasts from the SVZ to the striatum after stroke was reported (Arvidsson et al., 2002; Yamashita et al., 2006). Regional differences in the potential of SVZ cells are described, such as dorsolateral prevalence of oligodendroglial cells and neuronal and astroglial fates in the ventrolateral area (reviewed by Maki et al., 2013). In some cases, attraction of cells from the SVZ could not be shown by cell tracing experiments (Shimada et al., 2012) or fate mapping (Buffo et al., 2008). In contrast to the described promoting effects of stroke on the adult stem cell niche, chronic inflammation reduces proliferation and impairs migration of neuroblasts (Pluchino et al., 2008). So in general, local activation as well as an influence on the existing adult stem cell niches are conceivable and may take place in parallel. Certainly, this depends on the type, severity, and localization of the damage and further studies are needed to determine the contribution of both mechanisms in different lesion paradigms.

### Differences of the neurogenic potential *in vivo* and *in vitro*

In many cases, the neurogenic potential of the cells *in vivo* is more restricted compared to the situation *in vitro*. So reactive astrocyte-derived cells appear to be multipotent in culture, but fail to form neurons after transplantation *in vivo* (Shimada et al., 2012). An approach to promote the neuronal fate of reactive astrocytes is retroviral expression of the proneural transcription factor NeuroD1, allowing astrocytes to differentiate into glutamatergic neurons (Guo et al., 2014). Another transcription factor, Sox2, was able to convert spinal cord astrocytes into neurons (Su et al., 2014). A further strategy is the administration of neurogenesis-promoting factors, as shown for Galectin-1 after stroke (Ishibashi et al., 2007). More strategies have been summarized by Obermair et al. (2008).

The main difference between endogenous stem/progenitor cells *in situ* and their isolated and cultured counterparts is the completely changed environment, where signals from other cell types are lost. Among them are several neurogenesis-inhibiting factors (Seidenfaden et al., 2006; Buddensiek et al., 2009), one of the candidates is Notch (Aruga et al., 2002). Stress during isolation, high concentrations of growth factors in the medium, and the oxygen and energy supply are additional factors that may influence the cells’ potential. This shows that both, multipotent cells combined with a permissive environment, are necessary for the formation of neurons after lesion. The ECM contains a tremendous variety of signaling molecules and with regard to its importance for regeneration it is the topic of the next section.

## Extracellular signals influence regeneration and stem/progenitor cells

The ECM is a complex network of interacting molecules that are secreted by the cells into the extracellular space. Depending on the tissue, it functions as a scaffold for the cells and provides mechanical stability, for example in cartilage (Treilleux et al., 1992), but also in all other tissues. The ECM in the CNS is free of fibrillar elements, except after lesion (Heck et al., 2007). It contains glycoproteins like TN-C, TN-R, and proteoglycans (PG; Zimmermann and Dours-Zimmermann, 2008). PGs consist of a core protein and covalently attached carbohydrate (glycosaminoglycan, GAG) chains. Important Chondroitin sulfate proteoglycans (CSPGs) are molecules of the Lectican family and members of the RPTPβ family, whereas the Heparan sulfate proteoglycans (HSPGs) Syndecan and Glypican are other prominent constituents. Interactions of PGs with other molecules can be mediated by the core protein or by the carbohydrate structures. The ECM is able to bind growth factors and to present them to the cells (Clark, 2008; Brizzi et al., 2012). This allows the extracellular signals to regulate processes like cell survival, proliferation, and differentiation as well as migration or axon growth. According to changing requirements during development, the ECM composition is variable. After CNS lesions, the ECM is altered again. For example, reactive gliosis can lead to a glial scar that has a beneficial effect as it provides a barrier for healthy tissue from the environment (Pekny et al., 2014). At the same time, it is the major obstacle for axonal regrowth (Silver and Miller, 2004; Rolls et al., 2009). But the ECM is more than one constituent of the glial scar. It is involved in synaptic plasticity in many ways, for example in long term potentiation (LTP; Dityatev and Schachner, 2003). As mentioned above, the ECM contains signals regulating processes that are critical for regeneration and therefore this second section of the review is dedicated to details regarding the ECM composition and associated factors after CNS damage.

### Extracellular matrix after CNS damage

After lesion, several aspects of extracellular signals are critical: (i) barriers like the glial scar are important to protect the healthy tissue from the environment, but they also prevent axonal regrowth or cell migration; (ii) plasticity-limiting factors stabilize neuronal networks in the healthy brain but exacerbate reorganization in case of damage; and (iii) the balance between de-differentiation and re-differentiation. If cells are induced by the extracellular signals to de-differentiate, but get no signal for proper differentiation, they will stay in an undifferentiated state and cannot replace lost tissue. For example, TN-C, which is also present in the adult stem cell niche, may inhibit differentiation of an astrocyte-derived progenitor cell into a functional neuron or oligodendrocyte. This could be mediated by repression of the RNA-binding molecule Sam68 (Moritz et al., 2008; Czopka et al., 2010).

#### Extracellular matrix under different pathological conditions

Changes in the ECM composition differ depending on the type of CNS damage. As a disease-specific description is not the aim of this review, we recommend the following publications: stroke-induced effects were reviewed by Ellison et al. (1999), with focus on the blood-brain barrier by Baeten and Akassoglou (2011). Reports are also available for brain tumors (Gladson, 1999; Wade et al., 2013), spinal cord injuries (Condic and Lemons, 2002), neurodegenerative diseases like Alzheimer’s (Morawski et al., 2012), and for autoimmune disorders like multiple sclerosis (Sobel, 1998; van Horssen et al., 2007).

### Sources of extracellular matrix and associated signaling molecules

In response to damage, a number of cell types react and communicate via extracellular signals. The main sources of ECM and related important signaling molecules in the matrix are summarized in this paragraph. Important sources of extracellular molecules are listed in Table 1.

**Table 1:** **Important sources of extracellular signaling molecules after CNS lesion**.

**Cell type**	**Extracellular matrix-related molecule**
Endothelial cells	Brain-derived neurotrophic factor (BDNF)
	Fibroblast growth factor 2 (FGF-2)
	Leukaemia inhibitory factor (LIF)
	Pleiotrophin
	Vascular endothelial growth factor (VEGF)
	Fibrinogen [blood-derived]
Pericytes	Fibronectin
Astrocytes	Agrin
	Brevican
	Collagen IV, VIII
	Decorin
	Fibronectin
	Glypican
	Laminin
	Neurocan
	Phosphacan
	Syndecan
	Tenascin-C
	Thombospondin
	Versican
Oligodendrocytes/Precursors	Matrix metalloproteinase 9 (MMP-9)
	Neurocan
	Neuron-glial antigen 2 (NG2)
	Nogo-A
	Versican
Neurons	Chemokines
	Cytokines
	Sonic hedgehog (SHH)
Microglia/Macrophages	Cytokines
	Neuron-glial antigen 2 (NG2)
	Neurotrophic fawsctors

*As the origin of secreted molecules is often difficult to determine by immunological studies, more molecules are expressed that have not yet been allocated. References are given in the text*.

#### Vasculature

Blood vessels are an important signal source, as reviewed by Gattazzo et al. (2014). As part of the neurovascular niche they are also thought to be crucial for the residing stem/progenitor cells in the adult stem cell niches (Shen et al., 2008). Endothelial cells produce a number of factors that act on neural cells. Among others, Pleiotrophin, Leukaemia inhibitory factor (LIF), Brain-derived neurotrophic factor (BDNF), and Fibroblast growth factor (FGF) 2 influence differentiation (Carmeliet, 2003; Dugas et al., 2008). Vascular endothelial growth factor (VEGF) is an important regulator of angiogenesis and neurogenesis (Jin et al., 2002; Nag et al., 2002). Angiogenesis is increased in the damaged CNS and therefore more cells get in contact with the perivascular niche (Arai et al., 2009). Hypoxia supports stemness during development, but also under pathological conditions like stroke (Panchision, 2009). This is mediated by Hypoxia-inducible factor (HIF) I α, which facilitates Notch signaling and inhibits factors responsible for differentiation, for example Bone morphogenetic proteins (BMPs). The blood-brain barrier is disrupted in many cases as result of mechanical damage or chemokines (Dimitrijevic et al., 2006), which allows cells like T lymphocytes and additional factors from the blood to enter the CNS that are retained under normal conditions. When Fibrinogen from the blood crosses the disturbed blood-brain barrier, it activates astrocytes by providing Transforming growth factor β (TGF-β; Schachtrup et al., 2010). Pericytes are involved in the production of the glial scar, including Fibronectin (FN; Göritz et al., 2011). In addition, vessels are used as guiding structures by migrating neural progenitors in the healthy and diseased brain (Massouh and Saghatelyan, 2010).

#### Astrocytes

Astrocytes are extremely heterogeneous in their expression profile, as described in the first section. Depending on the type of damage, the time point after lesion, and the position, reactive astrocytes are responsible for TN-C expression in many lesion models (McKeon et al., 1991; Roll et al., 2012) and also express Brevican, Versican (Beggah et al., 2005), Neurocan (Haas et al., 1999), Phosphacan (McKeon et al., 1999), Decorin (Stichel et al., 1995), Laminin, and FN (Gris et al., 2007). HSPGs expressed by reactive astrocytes are Glypican, Syndecan, and Agrin (Iseki et al., 2002; Hagino et al., 2003; Falo et al., 2008). Collagen expression, which is involved in glial scar formation, was also shown for reactive astrocytes. The basement membrane-associated type IV (Liesi and Kauppila, 2002) and type VIII (Hirano et al., 2004) Collagens were detected. Astrocytes are also a source of Thrombospondins (TSPs; Lin et al., 2003) (reviewed by Sofroniew, 2009). Astrocytic expression of TN-C, Neurocan, and Phosphacan is increased by presence of meningeal fibroblasts *in vitro* (Wanner et al., 2008). Activation of astrocytes is mediated by pro-inflammatory cytokines like Interleukin (IL)-1β, IL-6, and Tumor necrosis factor α (TNF-α). They are secreted by microglia, in part by the astrocytes themselves, or by other cell types (Buffo et al., 2010).

#### Oligodendrocytes

Oligodendrocytes produce Nogo-A, an inhibitor of neurite outgrowth (Chen et al., 2000). Oligodendrocyte precursors express the CSPGs Neurocan and NG2 (Levine, 1994; Asher et al., 2000) and oligodendrocyte lineage cells secrete Versican under pathological conditions (Asher et al., 2002). Matrix metalloproteinase 9 (MMP-9) produced by oligodendrocyte precursors leads to blood-brain barrier leakage (Seo et al., 2013).

#### Neurons

Neurons start to express numerous chemokines like Chemokine (C-C motif) ligand 20 (CCL20), CCL21, CXC chemokine ligand 12 (CXCL12/SDF-1), and CXCL14/BRAK under pathological conditions (Das et al., 2012). They are primarily involved in immunomodulation, but additional functions as neuromodulators are assumed (Rostène et al., 2011).

#### Microglia

Microglia, the macrophages of the CNS, are activated by several mechanisms after injury. For example, they can detect reduced neuronal activity by alterations in neurotransmitter concentration and become activated (Biber et al., 2007; Kettenmann et al., 2011). Chemokines expressed after lesion can induce migration of microglia, for example Cysteine-cysteine (CC) chemokines (Carbonell et al., 2005). The consequence of microglial activation is the upregulation of cytokines and neurotrophic factors (Donnelly and Popovich, 2008). Increased levels of cytokines can induce remodeling of the ECM, for example by inducing the expression of MMPs (Gottschall and Yu, 1995) and of matrix molecules like TN-C by reactive astrocytes. Activated microglia also express ECM molecules themselves, for example NG2 (Sugimoto et al., 2014).

### Classes of extracellular matrix molecules in the postlesional CNS

**Figure 2:**
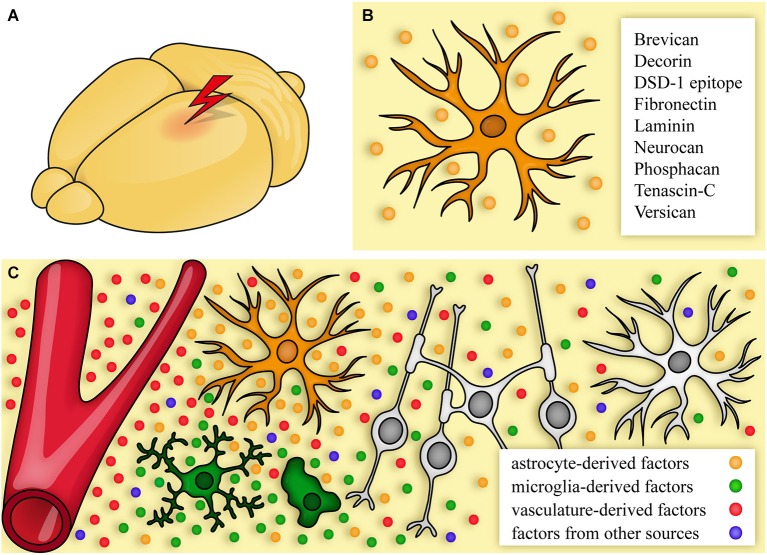
**The extracellular matrix after CNS damage. (A, B)** After lesion, cells start to produce an altered extracellular matrix (ECM). One major source are reactive astrocytes **(B)** that express CSPGs and other ECM molecules. **(C)** Signals come from astrocytes (orange), the vasculature (red), immune cells/microglia (green), and additional sources (blue, for a detailed list see Table 1). This environment can promote, but also inhibit regeneration by affecting neurons, astrocytes, and oligodendrocytes.

Important classes of extracellular signaling molecules that are typically present after CNS damage are described below. In many cases, components typical of the stem cell niches are re-expressed and provide an environment that resembles—in part—a neural stem cell niche like the SVZ. That niche is formed by signals from the vasculature, cerebrospinal fluid, and a complex set of extracellular molecules secreted by the cells in the niche (reviewed by Kazanis and ffrench-Constant, 2011). The situation after lesion with upregulated ECM molecules and the niche-like environment is summarized in Figure 2. As described above, major sources of the signals are reactive astrocytes, microglia, and the vasculature. Important functions regulated by ECM molecules and associated factors are listed in Table 2.

**Table 2:** **Parameters modulated by extracellular signals after lesion**.

**Parameter**	**Modulating factor in the extracellular matrix**
Cell survival, proliferation	Neurotrophic factors*
	Cytokines*
	Growth factors*
Differentiation, axon growth, synaptic plasticity	Agrin
	Brevican
	Collagen
	Decorin
	Glypican
	Laminin
	Neurocan
	Neuron-glial antigen 2 (NG2)
	Nogo-A
	Phosphacan; with attached DSD-1 epitope
	Pleiotrophin
	Syndecan
	Tenascin-C
	Tenascin-R
	Thrombospondins
	Versican
Migration	CXC chemokine ligand 12 (CXCL12)
	Fibronectin
	Laminin
	Tenascin-C
	Thrombospondins

*Many molecules regulate several aspects, only main functions are listed here. References are given in the text*.

* signaling is modulated by the extracellular matrix.

#### Chondroitin sulfate proteoglycans

CSPGs act as barrier after CNS damage and contribute to the axonal growth-inhibitory environment including the glial scar (Brown et al., 2012). CSPGs are mainly expressed by reactive astrocytes. Their effect can be mediated by members of the Leukocyte common antigen-related (LAR) family of RPTPs that comprises for example LAR, RPTPδ, RPTPσ, and the Nogo receptors NgR1 and NgR3 (reviewed by Cregg et al., 2014). LARs are involved in β-Catenin recruitment, leading to changes in the Actin cytoskeleton, which in turn affects axon growth and synaptic organization (Um and Ko, 2013). The Actin cytoskeleton is also targeted by NgR1 and coreceptors, in this case via the small GTPase RhoA and Rho-associated kinase (ROCK; Fournier et al., 2003; Schwab and Strittmatter, 2014). In addition to the above mentioned receptors, CSPGs can bind to specific receptors like Integrins, a family of heterodimeric transmembrane receptors (see the detailed Integrin description below). CSPGs can also act indirectly, when they intensify growth factor-induced signaling by presenting a growth factor to its receptor, or conversely, block signaling by sequestering growth factors like Pleiotrophin (Deepa et al., 2002) from their receptors (reviewed by Carulli et al., 2005; Sharma et al., 2012b). Neurocan, Brevican, and Versican levels are increased after lesion (Jones et al., 2003; Beggah et al., 2005) as well as Biglycan, Decorin (Stichel et al., 1995), and NG2 (Levine, 1994). NG2 inhibits neurite outgrowth, consequently its neutralization improves recovery (Dou and Levine, 1994; Petrosyan et al., 2013). It is produced by oligodendrocyte progenitors, but not by GFAP-positive astrocytes. This is in line with reports that NG2-positive progenitors are not an important source of astrocytes after brain injury (Komitova et al., 2011). Macrophages and microglia are additional sources of NG2 (Jones et al., 2002; Sugimoto et al., 2014). Decorin antagonizes scar formation as it is able to reduce the expression of Neurocan, Brevican, NG2, and Phosphacan, probably by inhibiting TGF-β and antagonizing the Epidermal growth factor (EGF) receptor (Davies et al., 2004). The RPTPβ (or “RPTPβ/ζ“) family comprises four isoforms. Alternative splicing of a single gene and proteolytic processing result in two transmembrane receptor forms (RPTPβ long and RPTPβ short) and two secreted molecules (Phosphacan and Phosphacan short isoform (PSI; Garwood et al., 2003; Chow et al., 2008)). The receptor forms contain two cytoplasmic tyrosine phosphatase domains, of which only one is catalytically active (Krueger and Saito, 1992). Substrates for this domain are β-Catenin, which is related to the Actin cytoskeleton and Wnt signaling (Meng et al., 2000), Fyn kinase (Pariser et al., 2005a), and others. Dimerization following Pleiotrophin binding inactivates the phosphatase domains and thereby increases the phosphorylation state of downstream factors like Adducin, a cytoskeletal protein (Pariser et al., 2005b). Other extracellular interaction partners are TN-C, Contactin/F3/F11, the cell adhesion molecules L1CAM and NCAM (Klausmeyer et al., 2007), and FGF (Milev et al., 1998b). RPTPβ isoforms are regulated after knife lesion of the cerebral cortex (Dobbertin et al., 2003), striatal stab wound (Barker et al., 1996), and after retinal laser lesion (Besser et al., 2009) and are critical for recovery (Harroch et al., 2002). PSI as the smallest member of the RPTPβ family promotes axon growth (Garwood et al., 2003). Two isoforms (RPTPβ long and Phosphacan) can be decorated with Chondroitin sulfate GAG chains. These chains regulate the affinity to binding partners, for example to Contactin/F3/F11 (Milev et al., 1998a). A specific Chondroitin sulfate structure, the “DSD-1” epitope, is recognized by the monoclonal antibody 473HD (Faissner et al., 1994; Gates et al., 1995). This epitope is expressed on neural stem cells during development (von Holst et al., 2006) and it has been shown that blocking of this epitope impairs neurosphere formation *in vitro*. Subpopulations of reactive astrocytes after laser lesion in the visual cortex of rats and mice express the DSD-1 epitope (Sirko et al., 2009; Roll et al., 2012). As mentioned above, this might reflect the immature characteristics of some reactive astrocytes and at the same time a neurogenic niche-like signal after CNS damage.

#### Collagens

Collagens comprise 28 members and can be divided into fibril-forming and network-forming Collagens (Ricard-Blum, 2011). In the CNS, Collagens are associated with the vasculature. Integrins are important Collagen receptors: the Integrin subunits α1, α2, α10, α11, and β1 interact with different Collagens. Additional receptors are members of the Discoidin domain receptors (DDRs), a subfamily of receptor tyrosine kinases (Leitinger and Hohenester, 2007). In addition to cell adhesion, Collagens are able to activate intracellular pathways and can induce proliferation (Pozzi et al., 1998). Collagens are expressed by astrocytes after lesion. Type IV and type VIII Collagens, both network-forming and associated with the basement membrane, have been found upregulated in the CNS (Liesi and Kauppila, 2002; Hirano et al., 2004). In general, Collagen expression is suppressed via EGF signaling (Heck et al., 2007).

#### Fibronectin

FN is a secreted glycoprotein that forms dimers via disulfide bonds near the C terminus. FN interacts with ECM molecules like Collagens, HSPGs, and TN-C and acts as ligand for Integrin receptors (Singh et al., 2010). FN is expressed by astrocytes and microglia/macrophages after CNS damage, also liver-derived plasma FN has a neuroprotective function in CNS repair (Tate et al., 2007a; Kim et al., 2013). FN activates microglia through binding to Integrin α5β1 (Milner et al., 2007) and has a neurite growth-promoting effect *in vitro* (Tom et al., 2004).

#### Heparan sulfate proteoglycans

HSPGs are, in contrast to the inhibitory CSPGs, ascribed to the neurite outgrowth-promoting factors (Yamaguchi, 2001). It is assumed that HSPGs can interfere with CSPG signaling by interacting with the same receptors, for example RPTPσ (Coles et al., 2011). Accordingly, downstream factors like N-Cadherin (Siu et al., 2007) might be regulated. The HSPG Glypican is expressed by reactive astrocytes (Hagino et al., 2003). It is involved in Hedgehog signaling (Filmus and Capurro, 2014) and as a HSPG it also regulates FGF signaling (Gordon et al., 1989; Rapraeger et al., 1991; Yayon et al., 1991). Syndecan, which is also able to modulate FGF signaling (Filla et al., 1998), is produced by astrocytes after brain injury (Iseki et al., 2002). Important for axonal growth and cell migration could be the interaction of Glypican-1 with repellent Slit proteins (Ronca et al., 2001). Slit is expressed by reactive astrocytes (Hagino et al., 2003), treatment with specific inhibitors of this interaction is owing (Lau and Margolis, 2010). The *Drosophila* homolog of Syndecan is involved in Slit signaling (Steigemann et al., 2004), suggesting a similar effect in the mammalian CNS. The HSPG Agrin is also expressed by reactive astrocytes. It has functions in the immune system and in neuromuscular synapse formation and may additionally play a role in CNS synapse formation during regeneration (Falo et al., 2008). Perlecan, which is associated with the vascular basement membrane, is a secreted HSPG that is upregulated following damage. After stroke, a neuroprotective effect was shown after administration of Perlecan domain V. An Integrin-mediated upregulation of VEGF in endothelial cells was attributed to the beneficial function (Lee et al., 2011).

#### Laminins

Laminins are trimeric glycoproteins and a crucial component of the basement membrane. Different isoforms of α, β, and γ chains are combined to at least 16 Laminins and interaction with other extracellular molecules like Agrin, Collagen IV, Nidogen, and Perlecan leads to formation of complex networks (Domogatskaya et al., 2012). Laminins signal through different Integrins (Belkin and Stepp, 2000) and promote cell adhesion, migration, and axon growth during development (Calof et al., 1994). Laminins are upregulated in the glial scar by astrocytes (Liesi et al., 1984; McKeon et al., 1995) and in vessels after lesion (Tate et al., 2007b; Sarkar et al., 2012). CSPG-mediated inhibition of neurite growth in the scar interferes with Laminin-induced promoting effects (McKeon et al., 1995). Migration of activated neural stem cells along vessels after injury seems to involve Laminin (Kokovay et al., 2010). This could be mediated by α6β1 Integrin, which is a well-known Laminin receptor and is expressed on neural stem cells (Shen et al., 2008).

#### Tenascins

The TN family comprises four members in vertebrates, namely TN-C, TN-R, TN-W, and TN-X (Chiquet-Ehrismann et al., 2014). In the nervous system, TN-C and TN-R are expressed (Joester and Faissner, 2001). TN-C is a glycoprotein that is expressed by astrocytes during development and in adult stem cell niches (Garcion et al., 2004; von Holst, 2008). It has a modular structure including EGF-like repeats and, as a result of alternative splicing, a variable number of FN Type III-like domains (Joester and Faissner, 1999, 2001; von Holst et al., 2007). As each domain is responsible for specific interactions with other extracellular molecules, TN-C isoforms have diverse functions. Six TN-C monomers form a hexamer, called “hexabrachion”. Expression of different TN-C isoforms is regulated by the transcription factor Pax6 (von Holst et al., 2007). TN-C interacts with CSPGs, for example with Neurocan (Rauch et al., 1997), members of the RPTPβ family (Barnea et al., 1994), as well as with Integrins (Yokosaki et al., 1998) and Contactin/F3/F11 (Rigato et al., 2002). Via its EGF-like repeats, TN-C also binds to the EGF receptor (Swindle et al., 2001). Interaction of TN-C has been shown for a number of Integrins, including αvβ3 (Jones and Jones, 2000a). TN-C-induced pathways include RhoA signaling and a Contactin-dependent inhibition of Fyn kinase. A model is proposed where inactive Fyn in turn prevents expression of the splicing factor Sam68 and thereby impairs oligodendrocyte differentiation (Czopka et al., 2010). TN-C blocks FN-dependent cell migration by interacting with Syndecan-4 (Chiquet-Ehrismann and Chiquet, 2003) and regulates axon growth (Faissner, 1997). During development, maintenance of oligodendrocyte precursors (Czopka et al., 2010) and astrocytic lineage progression depend on TN-C (Karus et al., 2011). After lesion, TN-C is re-expressed and it contributes to the glial scar as barrier (McKeon et al., 1991; Deckner et al., 2000). TN-C is detected in different regions of the lesioned CNS, for example in the hippocampus (Niquet et al., 1995; Nakic et al., 1996), cerebellum, and cortex (Laywell et al., 1992). TN-C expression was also reported in the human brain after traumatic brain injury (Hausmann and Betz, 2001). TGF-β and FGF2 stimulate TN-C expression (Smith and Hale, 1997). After lesion, TN-C isoforms containing the FN Type III-like domains B and D are strongly upregulated (Dobbertin et al., 2010). After spinal cord injury, TN-C has a beneficial effect on spinal cord recovery (Chen et al., 2010). An overview of the different functions of TN-C is given by Chiquet-Ehrismann et al. (2014). TN-R has also a modular structure like TN-C, but forms trimers instead of hexamers (Joester and Faissner, 2001). TN-R interacts with Integrins, for example β1 Integrins (Xu et al., 2014), Contactin/F3/F11, Phosphacan (Jones and Jones, 2000a), and Myelin-associated glycoprotein (MAG; Yang et al., 1999). TN-R is expressed after spinal cord injury (Apostolova et al., 2006) and in the lesioned optic nerve by an increased number of cells (Becker et al., 2000). TN-R is part of perineuronal nets (PNNs) that limit synaptic plasticity in the adult. After damage, this property seems to inhibit synaptic remodeling and thereby affects regeneration.

#### Thrombospondins

TSPs are secreted glycoproteins that form trimers (members of subgroup A) or pentamers (subgroup B) (Adams and Lawler, 2011). Interaction partners include ECM molecules like Laminins, Collagens, and PGs, growth factors like FGF2, and Integrin receptors that eventually trigger intracellular signaling cascades (Resovi et al., 2014). Thrombospondins are also involved in Notch signaling, which regulates astrocytic differentiation after lesion (Benner et al., 2013). TSPs are expressed by reactive astrocytes and microglia (Möller et al., 1996; Lin et al., 2003), for example they are upregulated after spinal cord injury (Wang et al., 2009b). TSPs are involved in oligodendrocyte precursor migration (Scott-Drew and ffrench-Constant, 1997), synapse formation (Christopherson et al., 2005), and angiogenesis inhibition (Armstrong and Bornstein, 2003).

#### Cell adhesion molecules

Cell adhesion molecules of the Immunoglobulin superfamily (IgSF CAMs) are membrane-bound receptors that mediate contact to the ECM and cell-cell interactions. Axon guidance is one important function of CAMs. Among them, Neural CAM (NCAM) is upregulated after CNS lesion and promotes spinal cord recovery (Zhang et al., 2010). Accordingly, recovery in NCAM knockout mice is affected. The polysialylated form of NCAM (PSA-NCAM) is associated with plasticity and is expressed during development, in the adult stem cell niche, and after CNS damage (Emery et al., 2003). NCAM can modulate GDNF and BDNF signaling (Vutskits et al., 2001; Nielsen et al., 2009) and interacts with the CSPG Neurocan (Friedlander et al., 1994). NCAM can activate a number of intracellular cascades, for example the mitogen-activated protein (MAP) kinase pathway via a complex of Spectrin, RPTPα, Fyn kinase and Focal adhesion kinase (FAK; Budinich et al., 2012). L1CAM, another member of IgSF CAMs, is also an important regulator of axon growth during development. It is again upregulated after lesions, where it limits corticospinal tract sprouting (Jakeman et al., 2006). L1CAM activates, in part together with Integrins, MAP kinase signaling via Src kinase and Phosphoinositide 3-kinase (PI3K). It also modulates the Actin cytoskeleton through Ankyrin and Spectrin and can recruit microtubules via Doublecortin (Maness and Schachner, 2007).

#### Integrins

Integrins, a group of heterodimer transmembrane receptors for ECM molecules, are involved in cell adhesion, axon growth, and numerous other processes. Each Integrin heterodimer consists of one α and one β subunit. Eighteen α and eight β subunits are described, leading to 24 confirmed Integrin heterodimers. Integrins connect ECM and the cytoskeleton via adapters like Talin and activate intracellular pathways that regulate gene expression. The classical downstream cascade of Integrins leads to activation of FAK and subsequent activation of Akt and MAP kinases (Guan, 1997; Guo and Giancotti, 2004). A FAK-independent activation of Src kinase also exists (Arias-Salgado et al., 2003). Some Integrins, including the subunits αv, β1, β4, and β7, can crosstalk with growth factor receptors like EGFR (Brizzi et al., 2012). The β1 subunit is important in stem cell biology (Campos, 2005). As already mentioned, Integrins bind a huge number of ECM molecules. For example, Integrin α6β1 binds Laminin (Shen et al., 2008) and αvβ3 binds TN-C (Jones and Jones, 2000b). In addition to these outside-in signaling pathways, Integrins can transduce inside-out signals. They are mediated by the cytoskeletal protein Talin and increase the affinity of Integrin for extracellular ligands (Anthis et al., 2009). Like their ligands, Integrins are regulated under pathological conditions and contribute to postlesional changes (Ellison et al., 1999).

#### Matrix metalloproteinases

MMPs are able to modulate the ECM by specific proteolytic cleavage (Candelario-Jalil et al., 2009). This function is important in the damaged CNS when the cells’ environment needs to be adjusted, for example to increase plasticity. MMP upregulation following injury is described for blood vessels, astrocytes, and microglia (Noble et al., 2002). MMPs degrade defined matrix components. So different TN-C isoforms are cleaved by specific MMPs (Siri et al., 1995), MMP-3 degrades the CSPGs Neurocan, Brevican, and Phosphacan (Muir et al., 2002). Effects of MMPs on regeneration have been described, for example of MMP-9 (Hsu et al., 2008). Here, migration of MMP-9-deficient astrocytes was reduced *in vitro*. MMP-9 is also involved in blood-brain barrier opening in the diseased CNS (Seo et al., 2013). The expression of MMPs in astrocytes can be regulated by the cytokines IL-1 and TNF-α (Gottschall and Yu, 1995). But not only migration is affected by MMPs: MMP-3 and MMP-9 play a role in neuronal differentiation in response to cytokines *in vitro* (Barkho et al., 2008).

#### Perineuronal nets

A specialized form of ECM are PNNs. They consist of Hyaluronan, TN-R, the CSPGs Aggrecan, Neurocan, and Brevican, and link proteins (Faissner et al., 2010; Ye and Miao, 2013). PNNs surround subpopulations of neurons, mostly Parvalbumin-positive GABAergic inhibitory interneurons, and are thought to stabilize synapses. Therefore their appearance is correlated with the end of a critical period during development. It has been shown that astrocytes play an important role in PNN formation, as they secrete components of the PNNs (Pyka et al., 2011). In line with this, the quadruple knockout of TN-C, TN-R, Neurocan, and Brevican either in astrocytes or hippocampal neurons leads to reduced PNN formation, accompanied by impaired synaptogenesis, synapse stability, and altered synaptic activity *in vitro* (Geissler et al., 2013). PNN degradation with the bacterial enzyme Chondroitinase ABC restored synaptic plasticity in the visual cortex after the critical period (Pizzorusso et al., 2002). Although single components like Neurocan can be upregulated after damage (Kwok et al., 2011), reduction of PNNs has been described, which is in line with the fact that plasticity is increased under this condition (Karetko-Sysa et al., 2011). This shows that not only the presence of molecules as such is important, but also the spatial distribution and the interaction of different factors.

### Growth factors and other signaling molecules in the matrix

#### Ephrins and Eph receptors

Ephrins and their counterparts, the Eph receptors, are potent regulators of neurite growth, as they are able to induce growth cone collapse. Activation of an Eph receptor tyrosine kinase following Ephrin binding leads to RhoA- and ROCK-induced changes of the Actin cytoskeleton (Wahl et al., 2000). Also bidirectional signaling is described, in this case Ephrins can activate RhoA and other pathways (Daar, 2012). Many Ephrins and Eph receptors are upregulated after CNS damage, as reviewed by Goldshmit et al. (2006). Ephrin-B2 and Eph-B2 seem to be involved in segregating invading fibroblasts at the glial scar (Bundesen et al., 2003).

#### Nogo-A

Nogo-A is a potent inhibitor of neurite outgrowth and interacts with a complex of receptors, including NgR1, that results in activation of ROCK signaling (reviewed by Schmandke et al., 2014). Nogo-A is produced by oligodendrocytes and is part of the myelin in the CNS. It is one important inhibitor of axonal regeneration (Chen et al., 2000). Interestingly, Nogo-A and NgR1 are also involved in the homeostasis of the adult SVZ. Here, proliferation and differentiation of neural stem cells are restricted by Nogo-A-expressing neuroblasts via NgR1. In addition, neuroblast migration is regulated by Nogo-A in an NgR1-independent mechanism that acts on ROCK (Rolando et al., 2012). Nogo-A and its receptor have been a target for several blocking experiments after stroke (Lee et al., 2004), spinal cord injury (Liebscher et al., 2005), and in other lesion models that showed beneficial effects of Nogo-A inactivation (reviewed by Overman and Carmichael, 2014; Schwab and Strittmatter, 2014). In intact animals, Nogo-A blocking showed no obvious side effects on cognitive function (Craveiro et al., 2013), although the Nogo-A receptor NgR1 is involved in memory formation (Karlén et al., 2009) and mice with downregulated Nogo-A show subtle cognitive deficits (Petrasek et al., 2014).

#### Semaphorins

Twenty Semaphorins are found in vertebrates, all of them contain a characteristic extracellular Sema domain. The most prominent function of Semaphorins is the regulation of axon growth and their ability to induce growth cone collapse. Semaphorins bind to Plexin and Neuropilin receptors. Different intracellular cascades eventually affect the cytoskeleton and, in many cases, induce growth cone collapse. Microtubule and Actin filament stability can be modified as well as gene expression (Neufeld and Kessler, 2008), pathways that depend on protein synthesis and independent mechanisms coexist (Manns et al., 2012). Sema3A is expressed by fibroblast-like cells after CNS injury (Pasterkamp et al., 1999) and in line with this, meningeal fibroblasts inhibit neurite outgrowth *in vitro* (Niclou et al., 2003). After optic nerve crush, short-term Sema3A upregulation was observed, whereas other class 3 Semaphorins were upregulated for days (Sharma et al., 2012a). L1CAMFc chimeric molecules can reverse the repellent effect of Sema3A, which shows that the combination of different factors is important rather than a single factor (Castellani et al., 2000).

#### Sonic hedgehog

Sonic hedgehog (SHH) is a signaling molecule that is able to regulate gene expression as well as the cytoskeleton. During development, it serves as morphogen and is involved in dorsoventral patterning. Binding of SHH to its receptor Patched relieves the repression of the seven-transmembrane receptor Smoothened. As a consequence, Gli transcription factors are activated and accumulate in the nucleus. This results in repression or activation of other transcription factors and altered gene expression (Fuccillo et al., 2006). Via a Gli-independent mechanism that involves a guanine nucleotide exchange factor (Tiam1) for the GTPase Rac1, SHH acts on the cytoskeleton during dendritic spine formation (Sasaki et al., 2010). SHH is secreted by neurons, endothelial cells (Sirko et al., 2013), and also by reactive astrocytes (Amankulor et al., 2009). This upregulation can only be observed under certain pathological conditions in the CNS. The expression differs depending on the type of damage. So it is detected after stab wound and stroke, but not in a murine Alzheimer’s model (Sirko et al., 2013). In this study, it was shown that SHH expression is necessary for multipotency of endogenous neural stem/progenitor cells. The HSPG Glypican, itself upregulated after damage, is involved in Hedgehog signaling (Filmus and Capurro, 2014).

#### Wnts

Secreted Wnt proteins influence gene expression and the cytoskeleton. The canonical Wnt pathway starts with binding of Wnt to Frizzled receptor and to the coreceptor LRP. Subsequently, a β-Catenin-degrading complex is inactivated and β-Catenin accumulates in the cell. It enters the nucleus, where it interacts with transcription factors and activates Wnt target genes (Reya and Clevers, 2005). Non-canonical Wnt pathways also affect the cytoskeleton (Semenov et al., 2007). Wnt proteins are upregulated after lesion and influence regeneration in different ways. It was shown that repulsive Wnts inhibit axonal growth in the spinal cord (Liu et al., 2008), on the other hand Wnt signaling is involved in symmetrical cell division of SVZ cells after stroke (Piccin and Morshead, 2011).

#### Notch

Notch is a transmembrane receptor that plays an important role in neural cell fate determination. After binding of its ligand, Jagged or Delta-like, the Notch intracellular domain (NICD) is cleaved by γ-Secretase. NICD translocates to the nucleus, where it interacts with transcription factors and allows target gene expression (Kopan and Ilagan, 2009). Notch is upregulated and activated after CNS lesion or stroke (Yamamoto et al., 2001; Xiao et al., 2009). Notch and its interaction with TSP 4 after cortical lesion are necessary for the differentiation of SVZ-derived astrocytes (Givogri et al., 2006; Benner et al., 2013). In another study, astrogliosis was promoted by the Notch ligand Jagged, produced by transplanted endothelial progenitor cells (Kamei et al., 2012).

#### Cytokines

Cytokines induce diverse cellular responses by activation of specific receptors. Examples for signaling cascades are reviewed by Leonard and Lin (2000) and Schlessinger and Ullrich (1992). Important cytokines expressed by activated microglia and macrophages are Interleukin-1 (IL-1), IL-6, Interferon, and TNF-α (Smith and Hale, 1997). TGF-β is increased in microglia under pathological conditions in the brain (Morgan et al., 1993) and the spinal cord (McTigue et al., 2000). They influence the expression levels of MMPs (see above) and activate astrocytes, IL-6 for example promotes EGF-induced astrocyte proliferation (Levison et al., 2000). Astrocytes are another major cytokine source (reviewed by Eddleston and Mucke, 1993; Wiese et al., 2012). BMPs and their inhibitor Noggin regulate neurogenesis in the adult stem cell niche (Lim et al., 2000). BMPs and Noggin are upregulated after brain and spinal cord injury (Hampton et al., 2007) and regulate astrocytic vs. neuronal and oligodendrocytic differentiation (Xiao et al., 2010). By driving the differentiation of astrocytes and CSPG production, BMPs are involved in glial scar formation (Fuller et al., 2007).

#### Neurotrophic factors

Neurotrophic factors and growth factors signal via specific transmembrane receptors. Depending on the class of growth factors, receptor tyrosine kinases (e.g., EGF receptor) or serine/threonine receptor kinases (e.g., TGFβ receptor) induce intracellular signaling cascades (Schlessinger and Ullrich, 1992). Neurotrophins like NT3 bind to Trk (Tropomyosin-related kinase) receptors (Hetman and Xia, 2000). Neurotrophic factors expressed by activated microglia and macrophages are BDNF, Ciliary neurotrophic factor (CNTF), Glial cell line-derived neurotrophic factor (GDNF), Hepatocyte growth factor (HGF), Insulin-like growth factor (IGF), Nerve growth factor (NGF), Neurotrophin (NT)-3 and Platelet-derived growth factor (PDGF; reviewed by Donnelly and Popovich, 2008). Microglia-produced neurotrophic factors can act in an autocrine way, for example BDNF and NT-3 increase microglia proliferation (Elkabes et al., 1996). In the adult spinal cord, high levels of CNTF are expressed following injury (Nakamura and Bregman, 2001). VEGF is secreted by endothelial cells and after damage also by astrocytes (Nag et al., 2002). It increases neurogenesis and plasticity (Jin et al., 2002; Licht et al., 2011). FGF and EGF increase levels of TGF-β (Lindholm et al., 1992), which is responsible for astrocyte activation and Neurocan expression (Asher et al., 2000). TGF-β increases NGF levels (Lindholm et al., 1990). NGF in turn induces microglial migration via the TrkA receptor, when combined with low concentrations of TGF-β (De Simone et al., 2007).

#### Chemokines

Chemokines are secreted signaling molecules that bind to G protein-coupled, seven-transmembrane receptors (Salanga et al., 2009). Activation of a receptor induces a cascade including second messengers like cAMP or IP_3_ that can mediate diverse cellular responses (Patel et al., 2013). The chemokine stromal-derived factor 1 (SDF-1/CXCL12) is expressed by astrocytes after stroke and mediates attraction of cells by binding to its receptor, CXCR4. This system is involved in attraction of bone marrow stem cells (Hill et al., 2004), transplanted umbilical cord blood cells (Rosenkranz et al., 2010), and neural stem cells (Imitola et al., 2004). Two isoforms, SDF-1α and SDF-1β, have distinct expression patterns in the lesioned CNS. Neurons express SDF-1α, whereas SDF-1β is expressed by endothelial cells (Stumm et al., 2002). Hence, a dual function of SDF-1-mediated modulation of the immune response and neurotransmission is discussed.

### Regeneration in ECM knockout (KO) models

The effect of ECM molecules on regeneration was examined in different knockout models. Double knockout of Neurocan and Brevican in mice does not obviously affect recovery after spinal cord injury (Quaglia et al., 2008). In a more sophisticated model with an additional priming lesion in the sensory nerve 7 days after spinal cord injury, only in KO animals axons crossed the dorsal root entry zone. This uncovered the growth-inhibiting effect of Neurocan and Brevican in the wild-type situation. TN-C KO mice have a mild phenotype, but recover worse after spinal cord injury (Chen et al., 2010). In contrast, deficiency of TN-R improves functional recovery of mice after spinal cord injury (Apostolova et al., 2006). TN-R seems to restrict synaptic reorganization after injury, which is in line with the role of TN-R in PNNs. EphA4 KO mice exhibit reduced gliosis and improved functional recovery after spinal cord injury (Goldshmit et al., 2004). Therefore, EphA4 seems to regulate two important aspects in regeneration.

### Extracellular matrix as target for improved recovery

Inhibitory matrix was the target of different approaches so far, a number of studies have been reviewed by Soleman et al. (2013). The function can be blocked with antibodies, specific inhibitors, by knockdown, or already synthetized ECM can proteolytically be degraded, for example by ADAM proteases (reviewed by Burnside and Bradbury, 2014). Inhibition of proteoglycan synthesis by β-D-Xylosides and sodium chlorate eliminates the inhibitory effect of astrocytic matrix on neurite growth *in vitro* (Smith-Thomas et al., 1995). Treatment with the Chondroitin sulfate-degrading enzyme Chondroitinase ABC (ChABC) revealed a recovery-promoting effect after spinal cord injury (Bradbury et al., 2002) and had neuroprotective effects (Chen et al., 2014). Although ChABC treatment is effective in many cases, ChABC-resistant inhibitory matrix also remains (Siddiqui et al., 2009) that needs to be addressed by other strategies. These inhibitory signals can be ascribed to the core protein part of PGs or to other molecules in the matrix. Treatment with antibodies directed against an extracellular factor relies on blocking its function. Studies using antibodies against Nogo-A (Zhao et al., 2013) and TN-R (You et al., 2012) have shown improved recovery from spinal cord injury. In addition to the aforementioned strategies that rely on the elimination of ECM function, delivery of regeneration-promoting ECM-related molecules is possible. For example, transplantation of L1CAM-overexpressing neural aggregates improved regeneration more effectively than those with normal L1CAM expression levels (Cui et al., 2011).

Possible mechanisms by which transplanted stem cells can promote regeneration in the context of ECM are discussed in the following third section.

## Potential effects of endogenous and transplanted stem cells on regeneration

As described in the first section, endogenous progenitor-like cells appear in the damaged CNS. If a subset of astroglial cells starts to de-differentiate anyway, what could be the benefit of additional, transplanted stem cells? It is clear that in many cases positive effects are not due to cell replacement (Chopp et al., 2009). The last section of this review gives an overview of the different strategies behind stem cell therapies.

**Figure 3:**
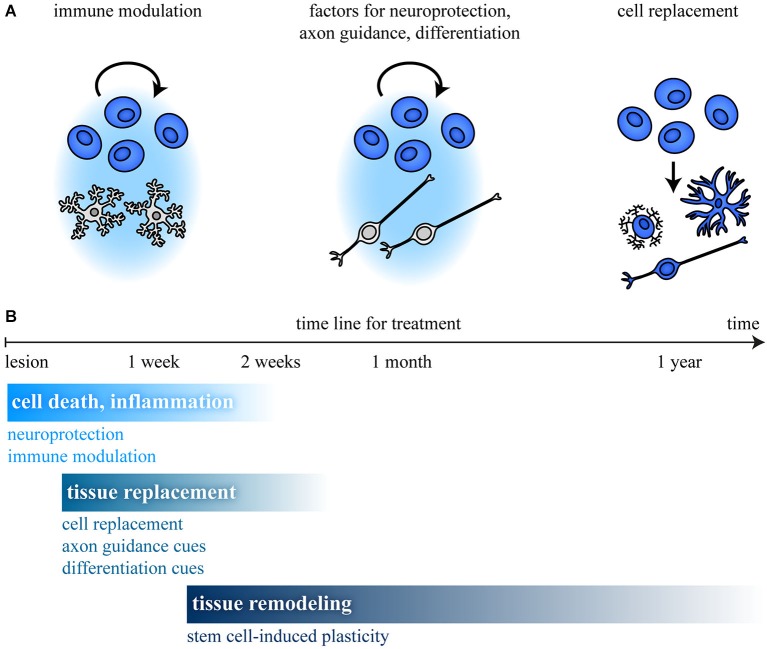
**Stem cell treatment and time line after CNS damage. (A)** Stem cell transplantation can promote recovery by different mechanisms. Modulation of (i) the immune response; (ii) production of factors that regulate survival, differentiation, or axon growth; and (iii) cell replacement are possible mechanisms. **(B)** In a first phase, cell death and inflammation (*ca*. 2 weeks) predominate, followed by a phase of tissue replacement (days to weeks) and tissue remodeling (months to years). This limits the time frame for efficient stem cell treatment, as shown for different aspects involved in regeneration. **(B)** Modified from Burda and Sofroniew (2014).

What are potential mechanisms by which transplanted stem cells support regeneration? Several strategies to improve recovery have been pursued so far (Burda and Sofroniew, 2014). Roughly the effects can be divided into three main aspects: (i) immune modulation; (ii) support of cell survival, differentiation, or axonal growth; and (iii) cell replacement (summarized in Figure 3A). Examples for factors that are produced by stem cells and their potential effects on regeneration are listed in Table 3. With regard to the phases of regeneration, where a first phase of cell death and inflammation (*ca*. 2 weeks) is followed by a phase of tissue replacement (days to weeks) and finally by tissue remodeling (months to years), at each time point different mechanisms have priority. This limits the time frame for efficient stem cell treatment (Figure 3B). Depending on the desired effect, stem cells of different origin and of different potential are the first choice.

**Table 3:** **Aspects potentially influenced by transplanted stem cells**.

**Parameter**	**Stem cell-released factor**
Immune system modulation	Chondroitin sulfate proteoglycans (CSPGs)
	Prostaglandin E
	Tenascin-C
Neuroprotection	Glial cell line-derived neurotrophic factor (GDNF)
	Nerve growth factor (NGF)
Axon growth, differentiation, synaptic plasticity	Matrix metalloproteinases (MMPs)
	Neurocan
	Phosphacan; with attached DSD-1 epitope
	Tenascin-C
	Tissue inhibitors of metalloproteinase (TIMPs)

*References are given in the text*.

The following aspects may account for differences of cultured and transplanted cells in comparison to the endogenous stem/progenitor pool: (i) the cell number of stem/progenitor cells may be higher when additional cells are transplanted; (ii) the potential of the cultured cells may differ from their endogenous counterparts due to the treatment (stress, addition of factors to the medium, etc.); (iii) not only the potential to differentiate may be changed during culture, but also the factors these cells secrete could differ depending on the pretreatment, the origin, or the differentiation state; (iv) transplantation of stem cells could modulate the immune system and therefore act indirectly on the CNS; and (v) stem cells can also be used as vehicle: for example, genetically modified stem cells stably deliver neurotrophic or other factors to damaged tissue, e.g., CNTF (Jung et al., 2013).

### Interaction of stem cells and the immune system

Immune cells can have both, positive and detrimental effects on regeneration, but also on the fate of endogenous and transplanted stem cells (reviewed by Martino et al., 2011; Kokaia et al., 2012). On the other hand, stem cells can modulate the immune response. For example, it has been shown that neurosphere-derived multipotent progenitors have a neuroprotective effect by immune modulation (Pluchino et al., 2005). Systemically transplanted NSCs improved recovery in a stroke model and reduced the expression of inflammation-associated genes (Bacigaluppi et al., 2009). Stem cells can also suppress T lymphocytes via increased nitric oxide and Prostaglandin E levels (Wang et al., 2009a).

In addition to the typical immune modulators mentioned above, molecules of the ECM can also influence the immune system: CSPGs do not only regulate axon growth, they are also able to modulate the immune response by binding to cytokines and Cluster of differentiation 44 (CD44; reviewed by Haylock-Jacobs et al., 2011). TN-C, a glycoprotein that is secreted by neural stem/progenitor cells *in vitro* (von Holst et al., 2007), can influence the immune system via Toll-like receptor (TLR) 4 (Midwood et al., 2009). TN-C and the immune system influence each other reciprocally: TN-C modulates the immune response, but is itself regulated by cytokines during inflammation. A detailed description is given in the review by Jakovcevski et al. (2013). Not only neural stem/progenitor cells can be utilized to improve recovery. For example, bone marrow stromal cells/mesenchymal stem cells (MSCs) reduce microglial activation (Yan et al., 2013). In addition to secreted factors, in the last years the role of exosomes in intercellular communication is being discussed (Ludwig and Giebel, 2012). These small vesicles loaded with lipids, proteins, and RNAs are released by MSCs and other cell types and might represent a mechanism by which transplanted cells can influence the immune system (Kordelas et al., 2014).

### Production of neurotrophic factors and regulation of plasticity

As described, stem cells can support neuronal survival by modulating the immune response, but they can also have a direct neuroprotective effect. Stem cells produce neurotrophic factors like GDNF and NGF (Lladó et al., 2004). Furthermore, they express a number of ECM molecules that are present in the developing nervous system. Among them are CSPGs like Neurocan, Phosphacan carrying the DSD-1 epitope (Ida et al., 2006; von Holst et al., 2006) and, as already mentioned, the glycoprotein TN-C. Some of these factors are well-known for their impact on cell differentiation, migration, and axon growth. If these factors are able to influence regeneration after transplantation will mostly depend on the number of surviving cells. A positive effect of human neural progenitor cells on axonal transport and plasticity after stroke was in part attributed to VEGF, TSP and Slit expression (Andres et al., 2011). Transplanted cells that express MMPs or they counterparts, the Tissue inhibitors of metalloproteinases (TIMPs), can control the ECM composition. Also non-neural cells can affect plasticity. So expression of MMPs and TIMPs was shown in MSCs, with differences in the expression profile depending on the origin of the cells (Lozito et al., 2014). MSCs induce SHH expression in host astrocytes that increases plasticity in conjunction with tissue-type plasminogen activator (tPA; Samson and Medcalf, 2006; Ding et al., 2013).

Plasticity can be induced not only on the molecular level. Neural precursors transplanted into the visual cortex differentiated into inhibitory neurons and formed synapses to host cortical neurons, thereby promoting plasticity after the critical period (Southwell et al., 2010).

### Neural differentiation and cell replacement by transplanted stem cells

Embryonic stem (ES) cells provide a source for the different cell types, because as pluripotent cells they can give rise to all cell types of the body, including neurons, astrocytes, and oligodendrocytes (Fraichard et al., 1995). To avoid the ethically problematic use of the embryo-derived ES cells, alternative sources for pluripotent cells were investigated. A promising alternative was described in 2006, when fibroblasts were reprogrammed to induced pluripotent stem cells (iPSCs; Takahashi and Yamanaka, 2006). The “Yamanaka cocktail” contains the four factors Oct3/4, Sox2, c-Myc, and Klf4. Subsequently, the number of factors could be reduced to two when adult neural stem cells were reprogrammed to iPSCs (Kim et al., 2008). Meanwhile, direct conversion of fibroblasts to induced neural stem cells (iNSCs) without a pluripotent state has been described (Han et al., 2012; Thier et al., 2012), reducing the risk of tumor induction by undifferentiated cells.

Cell fate decisions can be regulated, so astrocytic differentiation of transplanted NSCs is reduced when the CNTF is neutralized (Ishii et al., 2006). Astrocytes can be driven to a glutamatergic, NG2 glia to a glutamatergic and GABAergic neuronal cell fate by retroviral expression of the transcription factor NeuroD1 (Guo et al., 2014). Procedures for the directed differentiation into distinct neuronal subtypes exist, where cells are exposed to defined soluble and surface-bound factors. For example, glutamatergic (Zeng et al., 2010) and GABAergic (Maroof et al., 2013) neurons can be derived from ESCs or iPSCs. Also regional identities like retinal progenitors (Lamba et al., 2006) and subsequently photoreceptors can be produced (Lamba et al., 2010). But not only neurons, also oligodendrocytes as the myelinating cells of the CNS are important for regeneration and protocols are described that promote this cell fate (Neman and de Vellis, 2012). Functional integration of such cells was already shown, also for human cells (Tornero et al., 2013). A combined approach of stem cell-based treatment and manipulation of the ECM is the injection of neural stem cells with Chondroitinase ABC into the lesioned spinal cord (Ikegami et al., 2005). Axonal regrowth was significantly increased by the combined application.

## Summary/Outlook

As shown in this review, the CNS reacts to damage by upregulation of numerous extracellular signaling molecules that in part resemble the neurogenic stem cell niche. In line with this, endogenous stem/progenitor-like cells can be observed in many lesion models. The ECM contains important regulators of cell survival, differentiation, migration, or neurite outgrowth and is able to modulate signaling of associated molecules, for example of growth factors. These signals and the intrinsic properties of the cells present in the lesioned CNS are responsible for the outcome after regeneration. Stem cell transplantation now aims to influence this system or to add cells that replace lost tissue to a certain degree. Some approaches exploit neuroprotective effects by the stem cells, whereas others depend on cell replacement after differentiation and functional integration of the cells. The latter requires a carefully orchestrated sequence of complex actions and therefore seems to be harder to achieve. Stem cell transplantation experiments in animals will help to learn more about the molecular mechanisms that influence regeneration, as they allow to manipulate specific aspects in this complex interplay.

## Conflict of interest statement

The authors declare that the research was conducted in the absence of any commercial or financial relationships that could be construed as a potential conflict of interest.
